# Frequency of medical students' language expressing implicit uncertainty in simulated handovers

**DOI:** 10.5116/ijme.61e6.cde0

**Published:** 2022-02-25

**Authors:** Julia Gärtner, Sarah Prediger, Pascal O. Berberat, Martina Kadmon, Sigrid Harendza

**Affiliations:** 1 III. Department of Internal Medicine, University Medical Centre Hamburg-Eppendorf, Germany; 2 TUM Medical Education Centre, School of Medicine, Technical University of Munich, Germany; 3 Faculty of Medicine, University of Augsburg, Deanery, Augsburg, Germany

**Keywords:** Clinical reasoning, communication, handover, language, uncertainty, Germany

## Abstract

**Objectives:**

The aim of this study was to investigate the number and type of implicit expressions of uncertainty by medical students during simulated patient handovers.

**Methods:**

Eighty-seven volunteer medical students, a convenience sample collected on a first-come, first-served basis, participated in simulated handovers. They each worked with three simulated patients who presented with different chief complaints and personal conditions. The handovers were video recorded and transcribed. A framework of implicit expressions of uncertainty was used to identify and count modifiers that attenuate or strengthen medical information using MAXQDA lexical search. We analysed the findings with respect to the patients' contexts.

**Results:**

Implicit uncertainty expressions which attenuate or strengthen information occurred in almost equal frequency, 1879 (55%) versus 1505 (45%). Attenuators were found most frequently in the category 'Questionable', 1041 (55.4%), strengtheners in the category 'Focused', 1031 (68.5%). Most attenuators and strengtheners were found in the handover of two patients with challenging personal conditions ('angry man', 434 (23.1%) versus 323 (21.5%); 'unfocused woman', 354 (19.4%) versus 322 (21.4%)) and one patient with abnormal laboratory findings ('elevated creatinine', 379 (20.2%) versus 285 (18.9%)).

**Conclusions:**

Medical students use a variety of implicit expressions of uncertainty in simulated handovers. These findings provide an opportunity for medical educators to design communication courses that raise students' awareness for content-dependent implicit expressions of uncertainty and provide strategies to communicate uncertainty explicitly.

## Introduction

Dealing with uncertainty is one of the main challenges to be mastered in becoming a physician.^1^ Physicians rarely admit uncertainty^2^ and even tend to conceal it.^3^ Yet understanding and acknowledging uncertainty can facilitate professional behaviour.^4^ Physicians with higher tolerance of uncertainty appear to be better at addressing it.^4^ Verbal expressions of uncertainty are often used during decision-making and vary among different people and the context of the situation.^5^ The high between-subject variability of uncertainty expressions as well as their context-sensitivity can lead to misunderstandings.^5^ Additionally, residents are reported to feel uneasy inpatient encounters where diagnostic uncertainty persisted until the time of discharge because they had not formally been trained in communicating diagnostic uncertainty.^6^ 

A study of implicit uncertainty in primary care consultations showed an increased likelihood of expressing uncertainty when patients' symptoms were medically unexplained, particularly for diagnostic and treatment recommendations.^7^ Interestingly, no relationship between implicit expression of uncertainty and patient anxiety was found.^7^ The quality of medical student case presentations can also be improved by a clinical reasoning curriculum.^8^ Appropriate communication was found to improve the quality of handovers.^9^ Poor or conflicting handover communication reflects ambiguity about the patient's condition; this can have an impact on patient safety.^10^

Communication strategies during handovers are part of managing clinical uncertainty and influence the quality of information transfer.^11^ In a previous qualitative study, we developed a framework for the implicit expression of uncertainty from attenuators and strengtheners of information used in handovers during patient presentations.^12^ If uncertainty is not explicitly expressed or implicit expression of uncertainty goes unnoticed, it can become a threat to clinical decisions and patient safety. Becoming aware of implicit expressions of uncertainty will enable physicians and medical students to make uncertainty explicit and address it appropriately in order to prevent patient harm.

The aim of this study was to explore medical students' implicit expression of uncertainty during handovers in a simulated first day of residency by investigating the frequency of specific language, which can implicitly attenuate or strengthen the exchange of information. Because physicians express more uncertainty in inpatient cases with distracting contextual factors,^13^ the attenuating and strengthening expressions were individually investigated for all patient cases used in this simulation. Becoming aware of medical students' use of implicit expressions of uncertainty in different patient contexts will provide an opportunity for medical educators to establish communication courses to make medical students aware of how they use such expressions. This approach can support medical students' learning to detect their own uncertainty, which might otherwise go unnoticed, and will help them recognise specific contexts where their uncertainty arises. Additionally, such courses can provide strategies for the students to explicitly communicate uncertainty in clinical discussions in order to increase patient safety. 

## Methods

### Study design and participants

The study population was 87 medical students attending their final year of a 6-year undergraduate medical program at three medical faculties with different undergraduate curricula (Hamburg – vertically integrated curriculum, Oldenburg – vertically integrated curriculum in cooperation with Groningen University, and the Technical University of Munich – non-vertically integrated curriculum). Students were recruited in a convenience sample collected on a first-come, first-served basis after an e-mail invitation. The Ethics Committee of the Chamber of Physicians (Ethik-Kommission, Ärztekammer Hamburg), Hamburg, approved the study. Students were informed both orally and in writing about the purpose of the study and their rights as participants. Written informed consent was obtained from all participants. Participation was voluntary, and data were anonymised. The student’s mean age was 26.87 (SD = 3.59), 57 (65.5%) were female, 30 (34,5%) were male.

The recruited students participated in a 360-degree competence-based assessment simulating the first day of residency.^14^ The assessment included a consultation hour, where each participant saw three of six simulated patients, a patient management phase during which tests could be ordered, and a handover of the three patients to another participant. The students were randomly assigned to one of the two groups with three simulated patients each.^14^ The patient cases used in this study, which were portrayed by professional, trained actors, are summarised in Table 1. The patient cases, which were based on a chief complaint or finding, could only be solved by analytical reasoning and not by pattern recognition alone.^15^^,^^16^ The chosen chief complaints or main medical findings and the patients' personal condition represented specific contextual challenges in the daily medical routine.

**Table 1:** Simulated patient roles

SP	Sex	Age	Chief complaint	Condition	Diagnosis
1	M	46	Epigastric pain	Dissimulating	Soor esophagitis, HIV
2	M	44	Severe abdominal pain	Angry	Abdominal migraine, iatrogenic opioid dependency
3	F	46	Elevated creatinine level	Friendly	Acute renal failure due to hantavirus
4	M	52	Dull and constant abdominal pain	Taciturn	Chronic cholecystitis
5	F	35	Severe lower abdominal pain	Worried	Twisted ovarian cyst
6	F	48	General weakness	Unfocused	Vitamin B1 deficiency after gastric sleeve surgery

SP: simulated patient, M: male, F: female

### Instruments

To analyse the students' expression of implicit uncertainty during patient handover, we used our previously developed empirically derived framework^12^ based on data from our earlier 360-degree competence-based assessment.^17^ The development of this framework was based on qualitative research tools, which enabled the detection of implicit linguistic expressions of uncertainty in the appropriate context for it to be valid.^18^  Briefly, the framework provides categories of words or phrases that we identified as modifiers of information. The modifiers either attenuate or strengthen the stated information and, thereby, implicitly increase or decrease uncertainty. Attenuators which increased uncertainty consisted of the following categories, with subcategories in parentheses: 'Questionable' ('Questions', 'Hypothetical', 'Doubtful'), 'Incomplete' ('Inconclusive', 'Ambiguous', 'Unperceived', 'Absent'), 'Alterable' ('Indirectly modifying', 'Directly modifying' expression), and 'Unreliable' ('Expert opinion outside [specific medical] field', 'Lacking evidence'). Strengtheners, which implicitly decreased uncertainty consisted of these categories: 'Assertive' ('Instructions', 'Independently', 'Inevitable') 'Adequate' ('Coherent', 'Unambiguous', 'Perceptible'), 'Focused' ('Absolute', 'Simple', 'Prioritised'), 'Reliable' ('Medical expert opinion', 'Non-medical but insistent expert', 'Evidenced'). The complete framework depicting the language of different categories and subcategories of attenuators and strengtheners is described elsewhere.^12^

### Data collection methods

In 2018, the assessment described above took place in Hamburg, Germany. Every participant performed a handover of three patients, and the 261 handovers were videotaped. All videotaped patient handovers were transcribed verbatim. Data collection was performed by SP and SH.

### Data analysis

In 2020, JG searched the transcripts for words from all subcategories of the framework, which implicitly attenuate or strengthen information.^12^ This was done by using MAXQDA Analytics Pro 2020 (Release 20.0.8, VERBI GmbH). We excluded from the analysis the handover recipients' sentences and all sequences that were not related to the case presentation. Using MAXQDA's lexical search, all the words from the framework used by the case presenting student were identified, coded, and counted according to the framework's subcategories. In the search process, we used keywords as well as word combinations to ensure that no variants of the framework's modifiers were missed. We retrieved a frequency distribution of the modifiers within the frame' handover conversation' based on the absolute number counted by the MAXQDA Code-Relation-Browser.

## Results

While presenting their three patient cases, the 87 students used modifiers from our framework 3384 times in total; 1879 (55.5%) modifiers were identified as attenuators of information and 1505 (44.5%) modifiers as strengtheners of information. Tables 2 and 3 show the overall distributions of attenuators and strengtheners. Attenuators (Table 2) occurred most frequently in the category 'Questionable', 1041 (55.4%), and its subcategory 'Hypothetical' with the word 'maybe', e.g., "[...] maybe you need to run a CT scan […]". Strengtheners (Table 3) were most frequently detected in the category 'Focused', 1031 (68.5%), and its subcategory 'Absolute' with the word 'nothing' (e.g., "[...] there was [...] nothing else conspicuous […]."). Both Tables 2 and 3, show either one or two subcategories that accounted for at least 50% of the identified expressions. All attenuator subcategories occurred at least once, while for strengtheners, the subcategories' Independent' and 'Non-medical but insistent expert' were not detected.

Tables 4 and 5 show the overall and the patient-related distribution of the attenuators and strengtheners used when handing off the six different patients. The highest total numbers of attenuators (Table 4) were found for patient 2, 434 (23.1%), a 44-year-old angry man with severe abdominal pain (abdominal migraine), patient 3, 379 (20.2%), a 46-year-old friendly woman with elevated creatinine level (acute renal failure due to hantavirus), and patient 6, 364 (19.4%), a 48-year-old unfocused woman with general weakness(vitamin B1 deficiency after gastric sleeve surgery). The lowest total numbers of attenuators were identified for patient 5, 192 (10.2%), a 35-year-old worried woman with severe lower abdominal pain (twisted ovarian cyst) and patient 4, 219 (11.6%), a 52-year-old taciturn man with dull and constant abdominal pain (chronic cholecystitis). The attenuators category 'Questionable' showed the highest number of expressions for patient 2, 233 (22.4%). Attenuators of the category 'Incomplete' most frequently occurred during the handover of patient 6, 119 (23.2%). In the categories 'Alterable' and 'Unreliable', the highest number of attenuators was found for patients 2, 39 (22.3%) and 55 (36.4%), respectively.

**Table 2:** Distribution of identified attenuators in total and per category

Attenuators	Total		Category
	N	%	%
**Questionable**	**1041**	**55.4**	**100.0**
· Hypothetical	618	32.9	59.4
· Doubtful	370	19.7	35.5
· Questions (direct/indirect)	53	2.8	5.1
**Incomplete **	**512**	**27.2**	**100.0**
· Absent (finding/ experience/knowledge)	301	16.0	58.8
· Ambiguous	168	8.9	32.8
· Inconclusive	32	1.7	6.3
· Unperceived	11	0.6	2.1
**Alterable**	**175**	**9.3**	**100.0**
· Indirectly modifying	138	7.3	78.9
· Directly modifying	37	2.0	21.1
**Unreliable**	**151**	**8.1**	**100.0**
· Expert outside [specific medical] field	150	8.0	99.3
· Lacking evidence	1	0.1	0.7
**Total**	**1879**	**100.0**	**–**

**Table 3:** Distribution of identified strengtheners in total and per category

Strengtheners	Total		Category
	N	%	%
**Focused**	**1031**	**68.5**	**100.0**
· Absolute	486	32.3	47.1
· Prioritized	463	30.8	44.9
· Simple	82	5.4	8.0
**Assertive**	**231**	**15.3**	**100.0**
· Inevitable	143	9.5	61.9
· Instruction (direct/indirect)	88	5.8	38.1
· Independent	0	0.0	0.0
**Adequate**	**148**	**9.9**	**100.0**
· Unambiguous	71	4.7	48.0
· Perceptible	64	4.3	43.2
· Coherent	13	0.9	8.8
**Reliable**	**95**	**6.3**	**100.0**
· Evidenced	57	3.8	60.0
· Medical expert	38	2.5	40.0
· Non-medical but insistent expert	0	0.0	0.0
**Total**	**1505**	**100.0**	**–**

**Table 4:** Distribution of identified attenuators per patient

Attenuators	N	P1		P2		P3		P4		P5		P6	
		n	%	n	%	n	%	n	%	n	%	n	%
**Questionable**	**1041**	**164**	**15.8**	**233**	**22.4**	**216**	**20.7**	**135**	**13.0**	**97**	**9.3**	**196**	**18.8**
· Hypothetical	618	107	17.3	130	21.0	119	19.3	75	12.2	62	10.0	125	20.2
· Doubtful	370	48	13.0	93	25.1	86	23.2	51	13.8	27	7.3	65	17.6
· Questions (direct/indirect)	53	9	17.0	10	18.9	11	20.7	9	17.0	8	15.1	6	11.3
**Incomplete **	**512**	**80**	**15.6**	**107**	**20.9**	**112**	**21.9**	**45**	**8.8**	**49**	**9.6**	**119**	**23.2**
· Absent (finding/ experience/knowledge)	301	57	18.9	48	16.0	60	19.9	31	10.3	37	12.3	68	22.6
· Ambiguous	168	22	13.1	40	23.8	47	28.0	11	6.6	11	6.5	37	22.0
· Inconclusive	32	0	0.0	11	34.4	3	9.4	3	9.4	1	3.1	14	43.7
· Unperceived	11	1	9.1	8	72.7	2	18.2	0	0.0	0	0.0	0	0.0
**Alterable **	**175**	**25**	**14.3**	**39**	**22.3**	**35**	**20.0**	**18**	**10.3**	**34**	**19.4**	**24**	**13.7**
· Indirectly modifying	138	17	12.3	33	23.9	28	20.3	14	10.1	27	19.6	19	13.8
· Directly modifying	37	8	21.6	6	16.2	7	19.0	4	10.8	7	18.9	5	13.5
**Unreliable**	**151**	**22**	**14.6**	**55**	**36.4**	**16**	**10.6**	**21**	**13.9**	**12**	**7.9**	**25**	**16.6**
· Expert outside [specif. med.] field	150	22	14.7	55	36.7	16	10.7	21	14.0	11	7.3	25	16.6
· Lacking evidence	1	0	0.0	0	0.0	0	0.0	0	0.0	1	100.0	0	0.0
**Total**	**1879**	**291**	**15.5**	**434**	**23.1**	**379**	**20.2**	**219**	**11.6**	**192**	**10.2**	**364**	**19.4**

Legend: P1 (patient 1): 46-year-old dissimulating man with epigastric pain (soor esophagitis, HIV), P2 (patient 2): 44-year-old angry man with severe abdominal pain (abdominal migraine), P3 (patient 3): 46-year-old friendly woman with elevated creatinine level (acute renal failure due to hantavirus), P4 (patient 4): 52-year-old taciturn man with dull and constant abdominal pain (chronic cholecystitis), P5 (patient 5): 35-year-old worried woman with severe lower abdominal pain (torted ovarian cyst),P6 (patient 6): 48-year-old unfocused woman with general weakness (vitamin B1 deficiency after gastric sleeve operation)

**Table 5:** Distribution of identified strengtheners per patient

Strengtheners	N	P1		P2		P3		P4		P5		P6	
		n	%	n	%	n	%	n	%	n	%	n	%
**Focused**	**1031**	**158**	**15.3**	**232**	**22.5**	**176**	**17.1**	**130**	**12.6**	**99**	**9.6**	**236**	**22.9**
· Absolute	486	77	15.8	133	27.4	88	18.1	40	8.2	39	8.0	109	22.5
· Prioritized	463	70	15.1	76	16.4	76	16.4	77	16.6	53	11.5	111	24.0
· Simple	82	11	13.4	23	28.1	12	14.6	13	15.9	7	8.5	16	19.5
**Assertive **	**231**	**35**	**15.1**	**50**	**21.6**	**60**	**26.0**	**26**	**11.3**	**20**	**8.7**	**40**	**17.3**
· Inevitable	143	25	17.5	28	19.6	37	25.9	15	10.5	16	11.2	22	15.3
· Instruction (direct/indirect)	88	10	11.4	22	25.0	23	26.1	11	12.5	4	4.5	18	20.5
· Independent	–	–	–	–	–	–	–	–	–	–	–	–	–
**Adequate**	**148**	**21**	**14.1**	**26**	**17.6**	**28**	**19.0**	**26**	**17.6**	**21**	**14.1**	**26**	**17.6**
· Unambiguous	71	11	15.5	17	23.9	12	16.9	8	11.3	11	15.5	12	16.9
· Perceptible	64	8	12.5	8	12.5	13	20.3	16	25.0	9	14.1	10	15.6
· Coherent	13	2	15.4	1	7.7	3	23.1	2	15.3	1	7.7	4	30.8
**Reliable **	**95**	**5**	**5.3**	**15**	**15.8**	**21**	**22.1**	**11**	**11.6**	**23**	**24.2**	**20**	**21.0**
· Evidenced	57	3	5.2	5	8.8	16	28.1	5	8.8	19	33.3	9	15.8
· Medical expert	38	2	5.3	10	26.3	5	13.2	6	15.8	4	10.5	11	28.9
· Non-medical but insistent expert	–	–	–	–	–	–	–	–	–	–	–	–	–
**Total**	**1505**	**219**	**14.6**	**323**	**21.5**	**285**	**18.9**	**193**	**12.8**	**163**	**10.8**	**322**	**21.4**

Legend: P1 (patient 1): a 46-year-old dissimulating man with epigastric pain (soor esophagitis, HIV), P2 (patient 2): a 44-year-old angry man with severe abdominal pain (abdominal migraine), P3 (patient 3): 46-year-old friendly woman with elevated creatinine level (acute renal failure due to hantavirus), P4 (patient 4): a 52-year-old taciturn man with dull and constant abdominal pain (chronic cholecystitis), P5 (patient 5): 35-year-old worried woman with severe lower abdominal pain (torted ovarian cyst), P6 (patient 6): 48-year-old unfocused woman with general weakness (vitamin B1 deficiency after gastric sleeve operation)

The highest total numbers of strengtheners (Table 5) were found for the same patients as the highest numbers of attenuators, namely patient 2, 323 (21.5%), patient 3, 285 (18.9%), and patient 6, 322 (21.4%), while the lowest total number of expressions occurred in the handovers of patient 4, 193 (12.8%) and patient 5, 163 (10.8%). Strengtheners from the category 'Focused' were most frequently used in the handovers of patients 6, 236 (22.9%). Strengtheners from the categories' Assertive' and 'Adequate' were most often used during handovers of patient 3, 60 (26.0%) and 28 (19.0%), respectively, while strengtheners from the category 'Reliable' were most often used while handing over patient 5, 23 (24.2%).

## Discussion

Our study shows that the handovers in two of the simulated patients contained the most attenuators. These were two challenging patients: patient 2, a 44-year-old angry man with severe abdominal pain (abdominal migraine), and patient 6, a 48-year-old unfocused woman with general weakness (vitamin B1 deficiency after gastric sleeve surgery). These findings are consistent with other research showing that more uncertainty is expressed during clinical reasoning inpatient cases where contextual distractions are present.^13^^,^^19^ Physicians expressed the greatest uncertainty inpatient cases which contained contextual distractions that stemmed from either the patient (e.g., the complexity of presentation, English proficiency), the physician (e.g., fatigue, expertise, cognitive load), or the environment (e.g., length of consultation, the functionality of the electronic medical record).^13^

In our study, we also found the highest number of statements expressing implicit uncertainty inpatient cases where contextual distractions arose from the patients' physical condition or misleading symptoms, i.e., patient 2 with iatrogenic opioid dependence, patient 3 with a history of unnecessary gallbladder removal, and patient 6 with vitamin B1 deficiency after gastric sleeve surgery. Such distracting contextual elements of the presentation have been shown to elicit emotions that could influence the process of clinical reasoning by inducing uncertainty.^20^

For patient 5, a woman with a twisted ovarian cyst, and Patient 4, a man with chronic cholecystitis, the fewest attenuators of information were found. In these cases, the correct diagnosis is most frequently reported in the handovers. This could be related to these cases' less distracting or challenging emotional or medical contexts. In these two patients, the students seem to have developed a better internal representation of a suspected diagnosis, which is an important step towards confidence in clinical reasoning.^21^ This is supported by another study which showed that medical students' language also varied with the complexity of the patient cases.^7^ Our findings that the highest number of attenuators and strengtheners were associated with the same patients (patients 2, 3, and 6) are also consistent with the finding that higher uncertainty about a patient's diagnosis can lead to more descriptive detail.^22^

Overall, expressions that implicitly attenuated and strengthened information during patient handovers were used with very similar frequency, 1879 (55%) attenuators versus 1505 (44.5%) strengtheners. Among the attenuators, which imply increased uncertainty, the category 'Questionable' occurred most frequently. The word 'maybe' was the most frequently used single word from its subcategory 'Hypothetical'. In another study, within written radiology reports, 'maybe' was also identified as the most frequently used expression among all diagnostic certainty phrases (DCPs) which express uncertainty.^23^ How implicit DCPs are perceived also depends on the recipient. The interpretation of DCPs in radiology reports, for example, did not show good agreement between radiologists and primary care physicians with respect to the expressed level of uncertainty.^24^ Interestingly, good levels of agreement between radiologists and physicians from other specialities were found for DCPs expressing high levels of certainty, e.g., 'represents'.^25^ Possible sender-receiver differences with respect to words that implicitly express uncertainty underline the need to consciously become aware of such phrases and the effect they can have on others' perception of medical information. Presumably, the students in our study were not aware that they used attenuating and strengthening expressions that can imply increased or decreased uncertainty. It is also unclear in our study whether the receiving student perceived the attenuation or strengthening of the given information and how the degree of increasing or reducing uncertainty was decoded. Another study showed that student anxiety due to uncertainty was significantly higher in those who received the word 'hypothesis' in a patient handover compared to those who received the word 'diagnosis' for the same patient case.^26^

Attenuators from the category 'Questionable' and strengtheners from the 'Focused' category comprised 61.2% of all identified modifiers discovered in this study. This seems congruent with the two current meta-theories of truth related to clinical reasoning, which complement each other: 1) knowledge is true when it contains no inconsistencies and 2) truth is communicated as empirical accuracy.^27^ Interestingly, language from the category 'Questionable', e.g. 'maybe’,^7^ increase uncertainty in the clinical reasoning process because they indicate inconsistencies in the line of argument, while words from the category 'Focused', e.g. 'most likely’,^8^ decrease uncertainty by conveying accuracy. This reduces complexity, another mechanism in clinical reasoning to reduce uncertainty.^28^ Reducing uncertainty is one aim of clinical reasoning in physicians' daily practice,^29^ and evidence helps the reasoning process to make uncertainty manageable.^2^ In our study, expressions from the categories' Reliable' (strengthener) and 'Unreliable' (attenuator), both relating to sources of evidence quality, occurred least frequently. This might be because not all-important pieces of information had been acquired during history taking, or participants were still waiting for evidence in the form of laboratory results. Another hypothesis is that medical expertise could play a role in the number of expressions from certain attenuator or strengthener categories which could be related to the students' level of clinical reasoning, which differs from the clinical reasoning level of medical experts.^30^

Our study has several limitations. We used a convenience sample of participating students recruited on a first-come-first-served basis. This could mean a selection bias towards very good or interested students. The lexical search by MAXQDA based on the existing framework^12^ did not allow for any possible new relevant findings. However, this was not the intended aim of this study. Another limitation of this study is that the mere counting of expressions cannot measure combinations of strengtheners and attenuators. Furthermore, it should be noted that simple counting can neither do justice to the complex sender-receiver processes nor to cultural communication scripts such as politeness. A strength of this study is the high number of participating students from different medical schools as well as the standardised design of the patient cases and the structured training of the actors. The lexical search by MAXQDA enabled an efficient and focused language identification based on the framework's specific examples of implicit uncertainty expressions.

Further research should combine qualitative and quantitative methods to investigate combinations of strengthening and attenuating language. These studies would also ideally measure the handover recipient's perspective to explore degrees of implicit uncertainty and certainty phrases. Furthermore, the effect of language-culture needs to be considered as an important effect on the interpretation of these data.^31^ Since medical students rarely admit uncertainty^2^ and sometimes mask it,^3^ a practical application of this study would be for medical educators to raise medical students' awareness of expressions of uncertainty and the possible implicit effects of these on handovers. For example, the identification of a limited number of the most frequently used uncertainty phrases could be gathered by the use of artificial intelligence (AI) and used to provide student feedback. This would offer an opportunity for medical students to recognise and interpret implicit linguistic expressions of uncertainty and to learn how to make such uncertainty explicit, with a possible benefit of increased patient safety. Furthermore, it would be interesting to correlate the implicit expression of uncertainty in handovers with the information collected in medical history taking to correlate the medical quality of history taking with the expression of uncertainty in the handover.

## Conclusions

In conclusion, our study shows the variability and context-dependence of implicit expressions of uncertainty used by medical students in simulated handovers. Identifying students' implicit expressions of uncertainty can be used by medical educators in communication courses to increase medical students' awareness of their expressions of uncertainty. Furthermore, students should learn strategies to explicitly communicate uncertainty, especially in handover situations, where continuity of care and patient safety have top priorities. The handover presenter and the recipient both need to be aware of such implicit language and should instead explicitly discuss uncertainty. Future studies could address the question of whether such training can lead to more explicit expression of uncertainty, better clinical discussions, and eventually more favourable outcomes for the patients.

### Acknowledgements

We would like to thank all medical students who participated in this study. We thank Dr T. Urbanowicz for her support in preparing the transcripts. This study is part of the ÄKHOM-project, funded by the German Ministry of Education and Research (BMBF), reference number: 01PK1501A/B/C. Recipients of this grant were SH, POB, and MK. The funders had no role in study design, data collection and analysis, decision to publish, or preparation of the manuscript.

### Conflict of Interest

The authors declare that they have no conflict of interest.
